# Human myofiber‐enriched aging‐induced lncRNA *FRAIL1* promotes loss of skeletal muscle function

**DOI:** 10.1111/acel.14097

**Published:** 2024-01-31

**Authors:** Matthew J. Miller, Kevin J. Gries, George R. Marcotte, Zachary Ryan, Matthew D. Strub, Hawley E. Kunz, Bonnie K. Arendt, Surendra Dasari, Scott M. Ebert, Christopher M. Adams, Ian R. Lanza

**Affiliations:** ^1^ Division of Endocrinology Mayo Clinic Rochester Minnesota USA; ^2^ University of Iowa Iowa City Iowa USA; ^3^ Concordia University of Wisconsin Milwaukee Wisconsin USA; ^4^ Department of Quantitative Health Sciences Mayo Clinic Rochester Minnesota USA; ^5^ Emmyon, Inc. Rochester Minnesota USA

**Keywords:** aging, lncRNA, skeletal muscle

## Abstract

The loss of skeletal muscle mass during aging is a significant health concern linked to adverse outcomes in older individuals. Understanding the molecular basis of age‐related muscle loss is crucial for developing strategies to combat this debilitating condition. Long noncoding RNAs (lncRNAs) are a largely uncharacterized class of biomolecules that have been implicated in cellular homeostasis and dysfunction across a many tissues and cell types. To identify lncRNAs that might contribute to skeletal muscle aging, we screened for lncRNAs whose expression was altered in vastus lateralis muscle from older compared to young adults. We identified *FRAIL1* as an aging‐induced lncRNA with high abundance in human skeletal muscle. In healthy young and older adults, skeletal muscle *FRAIL1* was increased with age in conjunction with lower muscle function. Forced expression of *FRAIL1* in mouse tibialis anterior muscle elicits a dose‐dependent reduction in skeletal muscle fiber size that is independent of changes in muscle fiber type. Furthermore, this reduction in muscle size is dependent on an intact region of *FRAIL1* that is highly conserved across non‐human primates. Unbiased transcriptional and proteomic profiling of the effects of *FRAIL1* expression in mouse skeletal muscle revealed widespread changes in mRNA and protein abundance that recapitulate age‐related changes in pathways and processes that are known to be altered in aging skeletal muscle. Taken together, these findings shed light on the intricate molecular mechanisms underlying skeletal muscle aging and implicate *FRAIL1* in age‐related skeletal muscle phenotypes.

## Graphical abstract

FRAIL1 is a lncRNA that is elevated in skeletal muscle from older adults and negatively associated with muscle function. Forced expression of FRAIL1 in adult mouse muscle leads to decreased muscle fiber size and transcriptional and proteomic changes that recapitulate aging.

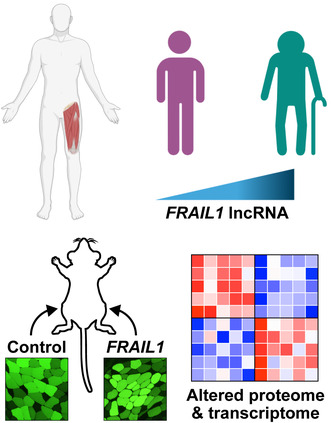

**Abbreviations**
GSEA: gene set enrichment analysis
GTEx: Genotype‐Tissue Expression
lncRNA: long noncoding RNAs
TA: tibialis anterior

## 1 INTRODUCTION

Aging‐associated skeletal muscle atrophy and functional decline (i.e., sarcopenia) is an important health concern in older individuals that is associated with an overall reduction in quality of life as well as numerous adverse health outcomes (Fielding et al., 2011; Goodpaster et al., 2006). Despite broad clinical significance, the molecular basis of age‐related muscle loss is not completely understood. Therefore, it is imperative to investigate the diverse molecular phenomena underlying age‐related skeletal muscle phenotypes to facilitate effective strategies to prevent, delay, or reverse this debilitating condition.

Long noncoding RNAs (lncRNAs) are a class of molecules that have been increasingly recognized as relevant to the regulation of skeletal muscle mass during aging and other conditions that promote skeletal muscle wasting (Liu et al., 2021; Nie et al., 2015). LncRNAs are greater than 200 nucleotides in length and, despite being actively transcribed and epigenetically regulated, are apparently not translated into proteins (Statello et al., 2021). Across nearly all tissues, lncRNAs have been implicated in the regulation of gene expression at epigenetic, transcriptional, posttranscriptional, and translational levels (Oo et al., 2022; Statello et al., 2021). In the context of age‐related skeletal muscle atrophy, specific lncRNAs have begun to emerge as critical regulators. For example, the aging‐associated cardiac and skeletal muscle‐enriched transcript *Chronos* has been found to negatively regulate skeletal muscle mass through Ezh2‐mediated suppression of *Bmp7* gene expression (Neppl et al., 2017). Additionally, *Cytor*, an exercise‐induced lncRNA negatively regulated during normal aging, induces a fiber‐type shift to a fast muscle phenotype by binding and sequestering the TEAD1 transcription factor (Wohlwend et al., 2021). These examples highlight the significance of lncRNAs in shaping the molecular landscape of skeletal muscle physiology. In contrast to these examples, which display functionally relevant conservation between humans and rodents, the primary nucleotide sequence of most human lncRNAs is often poorly conserved across taxonomic phyla beyond primates (Johnsson et al., 2014; Kapusta & Feschotte, 2014; Pang et al., 2006; Washietl et al., 2014), which presents a significant barrier to their study in mature skeletal muscle fibers using model organisms. Furthermore, unlike proteins that possess well‐established functional domains, the characteristic functional sequences of lncRNAs have not been fully explored (Statello et al., 2021).

In this study, we identify an understudied, but highly expressed, human skeletal muscle‐enriched lncRNA transcript termed *FRAIL1* (myoFiber‐enRiched Aging‐Induced Long non‐coding RNA 1) that is induced during normal aging alongside diminished muscle function. To this point, investigations of *FRAIL1* have been limited to one previous study, which described *FRAIL1* as among the top 10 human skeletal muscle RNAs most correlated with normal aging (Tumasian III et al., 2021). Here, we find that ectopic expression of *FRAIL1* in mature mouse skeletal muscle fibers is sufficient to induce muscle atrophy. Furthermore, we utilize mouse skeletal muscle as a model system to characterize the cellular effects of *FRAIL1* expression in mature skeletal muscle fibers in vivo. To this end, we performed unbiased transcriptomic and proteomic profiling of mouse skeletal muscle expressing *FRAIL1* and revealed widespread changes in components regulating muscle mass and function. The results of this study implicate *FRAIL1* in human aging‐associated skeletal muscle functional decline.

## 2 RESULTS

### 2.1 *FRAIL1* is a highly abundant aging‐induced skeletal muscle lncRNA

**FIGURE 1:**
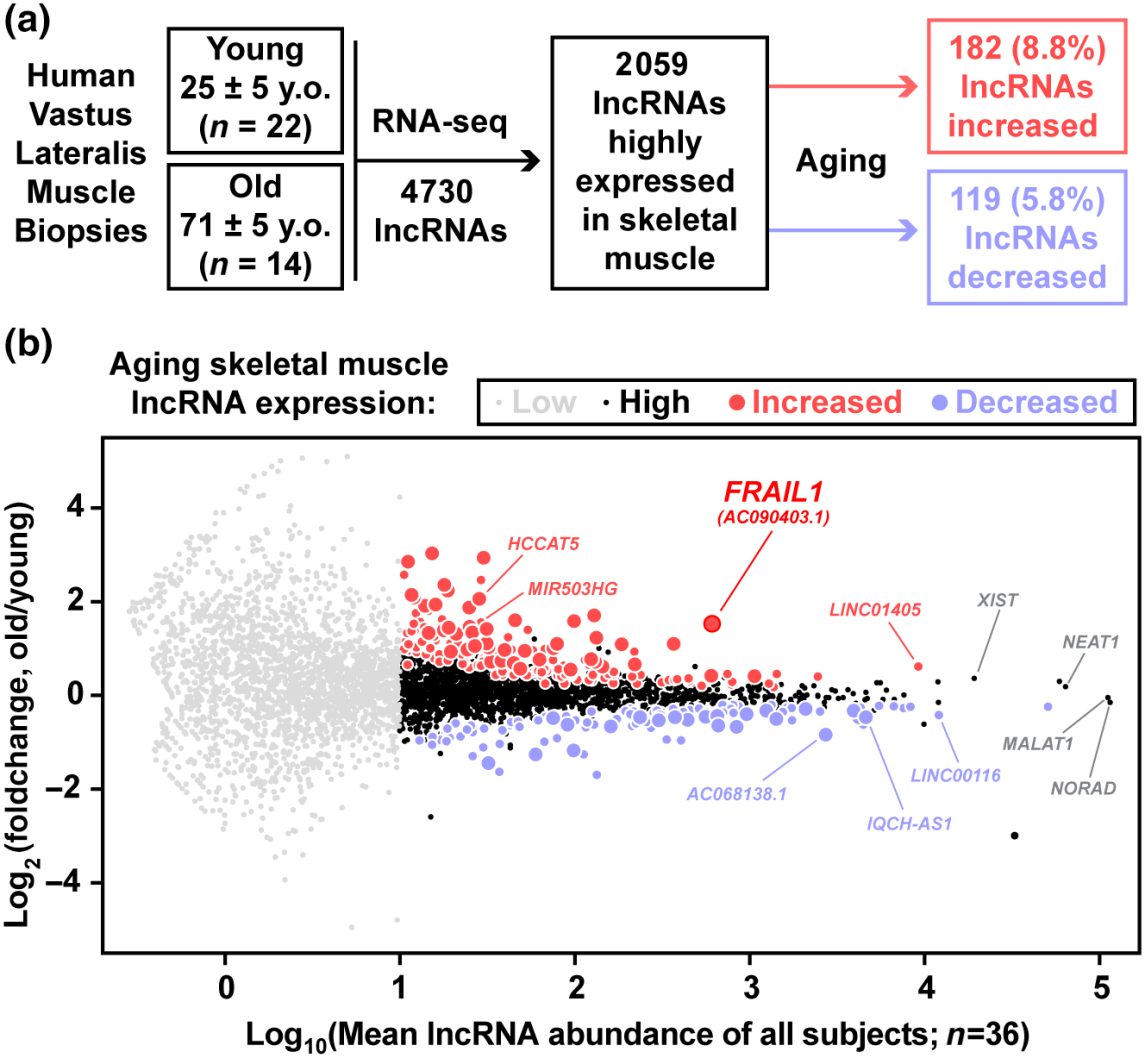
*FRAIL1* is a highly abundant skeletal muscle lncRNA induced by aging. (a) Overview of the study design and strategy used for identification of differentially expressed human lncRNA transcripts during skeletal muscle aging. RNA‐sequencing data from a previous study were filtered with high stringency and analyzed for lncRNA expression in skeletal muscle (average normalized RNA‐Seq counts across all samples ≥10) and differential expression between aged and young samples. (b) MA plot (log‐intensity ratios (*M* values, *y*‐axis) versus log‐intensity averages (*A* values, *x*‐axis)) of differences in lncRNA abundance between aged and young samples and average lncRNA abundance across all samples. Gray symbols represent lncRNA transcripts that were expressed below the abundance cutoff, black symbols represent skeletal muscle‐expressed lncRNAs that did not change with age, red circles are increased transcripts, and blue circles are decreased transcripts (smaller circles *p* < 0.05; larger circles Benjamini‐Hochberg corrected FDR <0.1).

We identified lncRNAs that are highly induced during aging in human skeletal muscles using RNA‐Seq of skeletal muscle biopsies obtained from young (25 ± 5 years; *n* = 22) and older (71 ± 5 years, *n* = 14) donors. A total of 2059 lncRNAs were robustly expressed in human skeletal muscle, and age‐related differences were evident in 182 lncRNAs (8.8%) that increased with age and 119 lncRNAs (5.8%) that decreased with age (Figure 1a, Table S1). Among the most highly expressed lncRNAs across all samples were the well‐characterized lncRNA transcripts *NORAD* (Soghli et al., 2021), *MALAT1* (Zhang et al., 2017), and *NEAT1* (Taiana et al., 2020), which did not differ in expression levels between young and old muscle samples (Figure 1b). Skeletal muscle lncRNAs downregulated by aging included *AC068138.1* and *IQCH‐AS1*, both of which are also repressed in thyroid cancer where low levels are associated with poor prognosis (Fei et al., 2023; Guo et al., 2019; Yuan et al., 2020). Skeletal muscle lncRNAs upregulated by aging included *HCCAT5*, a hepatocellular carcinoma‐associated transcript (Liu et al., 2010, 2013), and *MIR503HG*, which has been implicated in endothelial‐to‐mesenchymal transition in vascular disease (Monteiro et al., 2021) (Figure 1b). The vast majority of aging‐responsive differentially expressed skeletal muscle lncRNAs have not undergone in‐depth examination and remain poorly understood.

One of the most abundant differentially expressed lncRNAs between old and young individuals was *FRAIL1*, which was upregulated approximately 3‐fold in muscle tissue of older adults (Figure 1b). Despite being largely un‐studied, this transcript exists in nearly all public databases and is known by a series of alternative identifiers (*AK127888.1*, *RP11‐739 N10*, *AC090403*, *ENSG00000264151*, and *ENST00000584546*). The *FRAIL1* gene encodes a five‐exon spliced 1352 nucleotide lncRNA transcript that is located on human chromosome 18 in the intergenic region between the protein‐coding genes *CHST9* and *CDH2* (Figure S1A). Interrogation of human RNA‐Seq alignments showed that all five exons of *FRAIL1* are expressed in skeletal muscle with sequencing reads spanning the annotated splice junctions. Consistent with its classification as a lncRNA, in silico determination of coding potential indicated that *FRAIL1* is not predicted to be peptide coding (Figure S1B; Wang et al., 2013). Interestingly, a recently published study of the skeletal muscle transcriptome during normal aging identified *FRAIL1* as the only lncRNA within the top 10 skeletal muscle RNAs most positively correlated with age (Tumasian III et al., 2021). These findings identified *FRAIL1* as among the most highly expressed, aging‐induced lncRNAs in human skeletal muscle.

### 2.2 *FRAIL1* is enriched in skeletal muscle of healthy older adults

**FIGURE 2:**
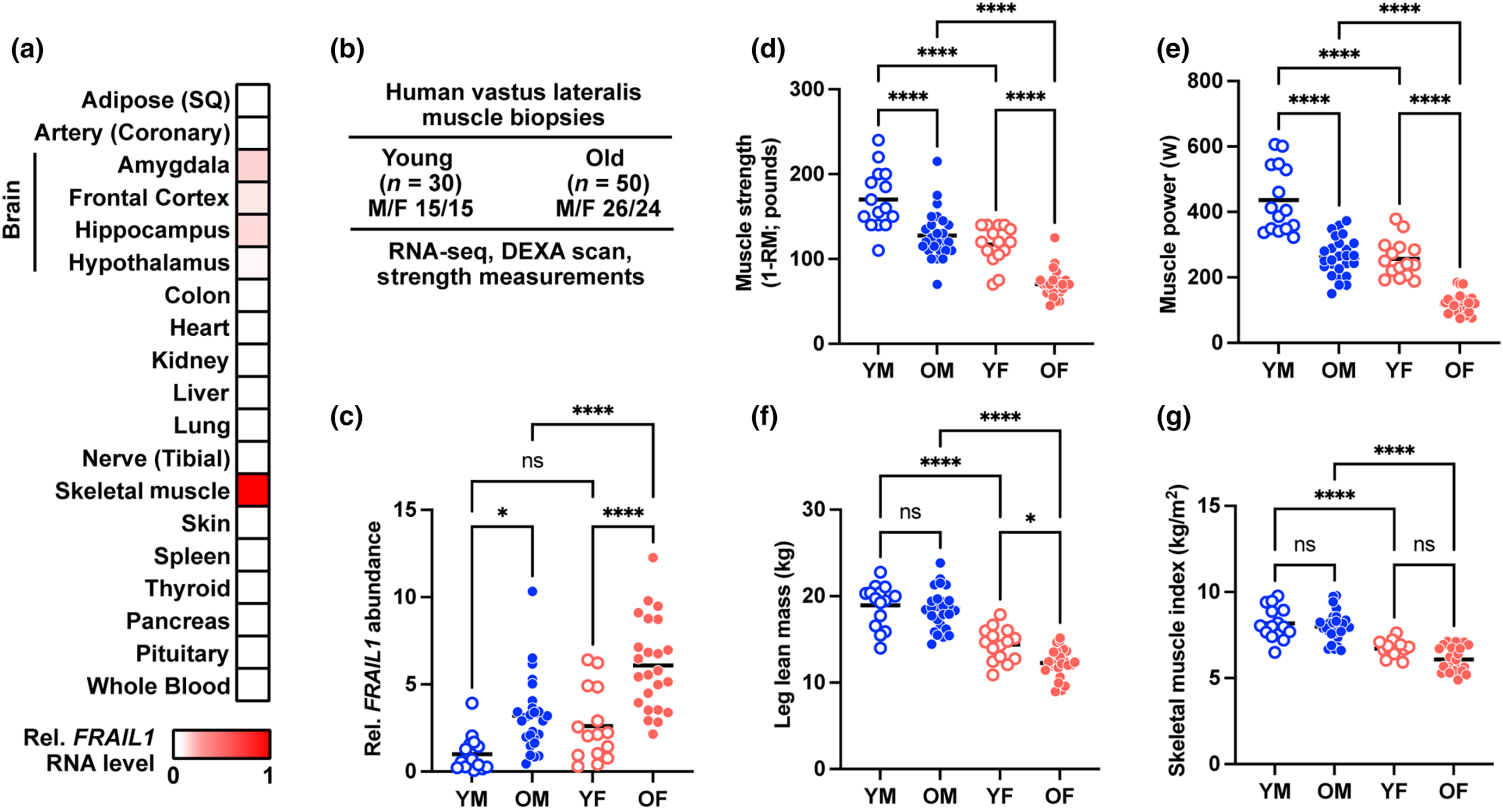
FRAIL1 is skeletal muscle‐enriched and associated with characteristics of aging muscle. (a) GTEx bulk tissue expression levels of FRAIL1 lncRNA expressed as the median TPM value for each human tissue type relative to the expression level in skeletal muscle. (b) Overview of human study participant characteristics and phenotypic measurements. (c) Relative skeletal muscle FRAIL1 lncRNA level across study participants. Data are organized by age group and sex and normalized to the average FRAIL1 lncRNA level in young males. *p* Values were determined by one‐way ANOVA. (d–f) Age‐ and sex‐specific muscle function outcomes and muscle mass estimates maximal knee extension muscle strength (d), peak knee extension power (e), leg lean mass (f), and skeletal muscle index (g) in young males (YM), young females (YF), older males (OM), and older females (OF). *p < 0.05, ****p < 0.0001.

To investigate the tissue‐specificity of *FRAIL1* lncRNA, we utilized publicly available human RNA‐Seq data from the Genotype‐Tissue Expression (GTEx) consortium (GTEx Consortium, 2013). This analysis revealed that *FRAIL1* is highly enriched in skeletal muscle. Importantly, *FRAIL1* was not detectable in other tissues, including cardiac muscle, with trace levels in several brain regions (Figure 2a). Apart from skeletal muscle, the only tissue that exhibited notable *FRAIL1* lncRNA expression was the testis (Figure S2). This observation is consistent with the high complexity of the testis transcriptome, which displays a higher extent of genome‐wide transcription than other organs (Hong et al., 2018; Soumillon et al., 2013). Analysis of GTEx single nucleus RNA‐Seq (snRNA‐Seq) data revealed that within human gastrocnemius muscle, *FRAIL1* was most enriched within myonuclei, indicating that *FRAIL1* is predominantly expressed within skeletal muscle fibers (Figure S2). To further profile skeletal muscle *FRAIL1* expression between ages and sexes and to characterize its relationship with muscle mass and function, we evaluated RNA‐Seq data from a separate, independent cohort of human skeletal muscle biopsies obtained from healthy young (27.0 ± 4.11 years; *n* = 30) and old (71.4 ± 4.7 years, *n* = 50) males and females (Figure 2b). Here, *FRAIL1* expression was the lowest in young muscle, with higher expression in females relative to males within both age groups (Figure 2c). In both males and females, muscles from older adults exhibited a 3‐ to 4‐fold increase in skeletal muscle *FRAIL1* abundance relative to their young counterparts, with the highest levels of *FRAIL1* observed in older females (Figure 2c). The increased FRAIL1 abundance in muscle from older adults was accompanied by decreased knee extensor muscle strength (Figure 2d) and decreased peak power (Figure 2e). Leg muscle mass, estimated from leg lean mass (Figure 2f), and skeletal muscle index (Figure 2g) were not remarkably different between young and older adults.

**FIGURE 3:**
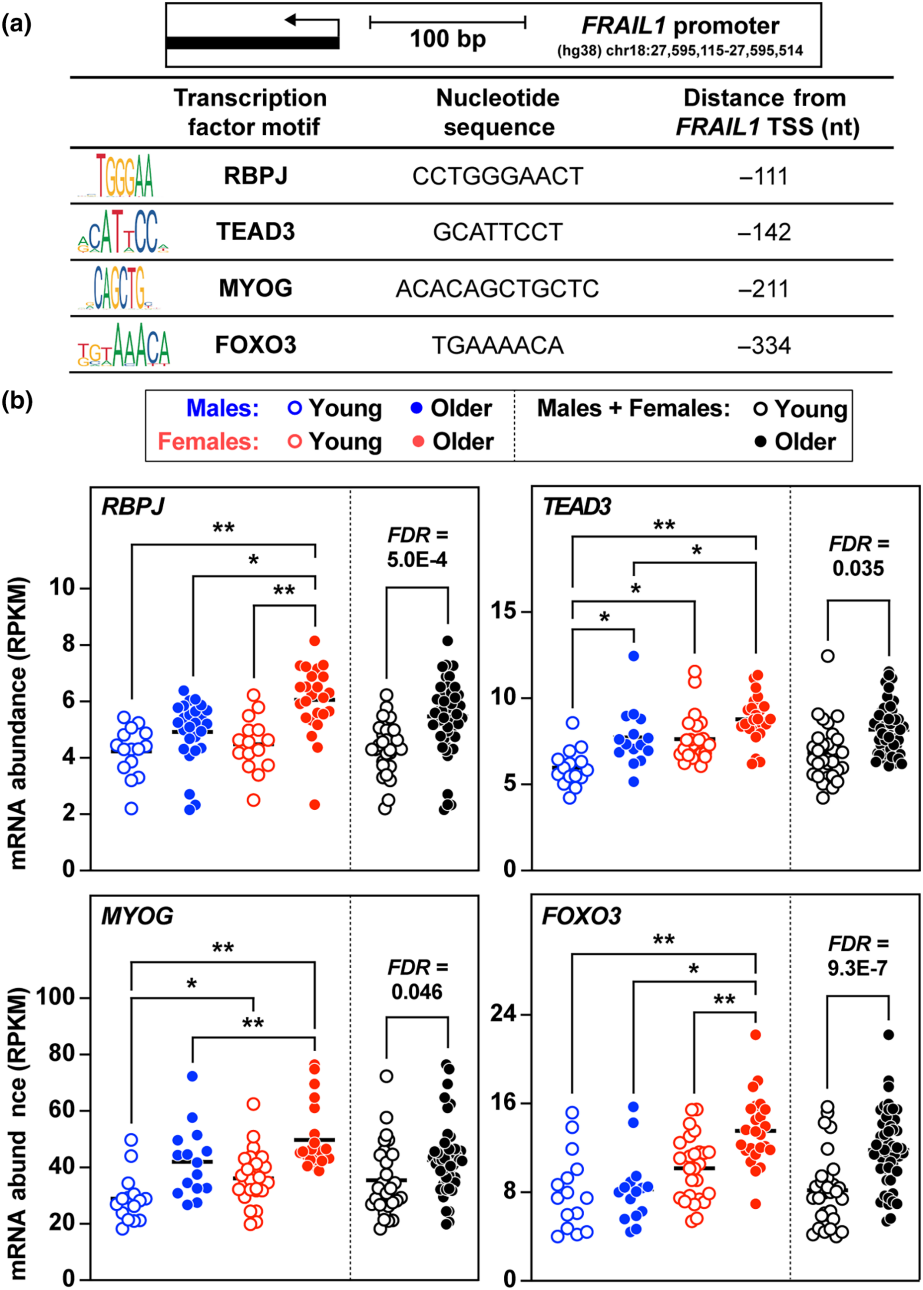
The transcription factors RBPJ, TEAD3, MYOG, and FOXO3 potentially regulate *FRAIL1* expression. (a) The JASPAR database was queried for transcription factor binding sites located within the promoter region 350 nucleotides upstream of the *FRAIL1* transcription start site. (b) Human vastus lateralis abundance levels of mRNAs encoding the transcription regulators RBPJ, TEAD3, MYOG, and FOXO3. Data are organized by age group and sex. For a comprehensive list of identified transcription factor binding sites and expression levels, see Figure S3. Horizontal bars indicate mean values from each group, false discovery rate (*FDR*) was determined by Benjamini‐Hochberg correction, and *p* values were determined by one‐way ANOVA. *p < 0.05, **p < 0.001.

To identify mechanisms that may regulate *FRAIL1* expression during skeletal muscle aging, transcription factor binding sites within the *FRAIL1* promoter region were interrogated using the JASPAR database (Castro‐Mondragon et al., 2022). This analysis revealed the presence of DNA sequence motifs that bind numerous transcription factors that are expressed in skeletal muscle (Figure 3a, Figure S3A). Across the same age and sex groups mentioned above, several transcription factors identified were robustly expressed, exhibiting sex‐ and age‐dependent differences in mRNA expression that mirrored differential *FRAIL1* expression. Notably, mRNAs encoding the transcription factors RBPJ, TEAD3, MYOG, and FOXO3 significantly increased with age and tended to be more highly expressed in older female skeletal muscle relative to males (Figure 3b, Figure S3B). This suggests that these transcriptional regulators, which have been previously implicated in skeletal muscle health (Gioftsidi et al., 2022; Joshi et al., 2017; Macpherson et al., 2011; Ninfali et al., 2018; Reed et al., 2012; Vasyutina et al., 2007; Zammit, 2017), may contribute directly to *FRAIL1* expression in skeletal muscle fibers. These findings identified *FRAIL1* as a skeletal muscle‐enriched, and potentially transcriptionally regulated, lncRNA whose abundance is associated with age‐related reductions in lean mass and muscle function in humans.

### 2.3 Forced expression of *FRAIL1* in mouse skeletal muscle causes muscle atrophy

**FIGURE 4:**
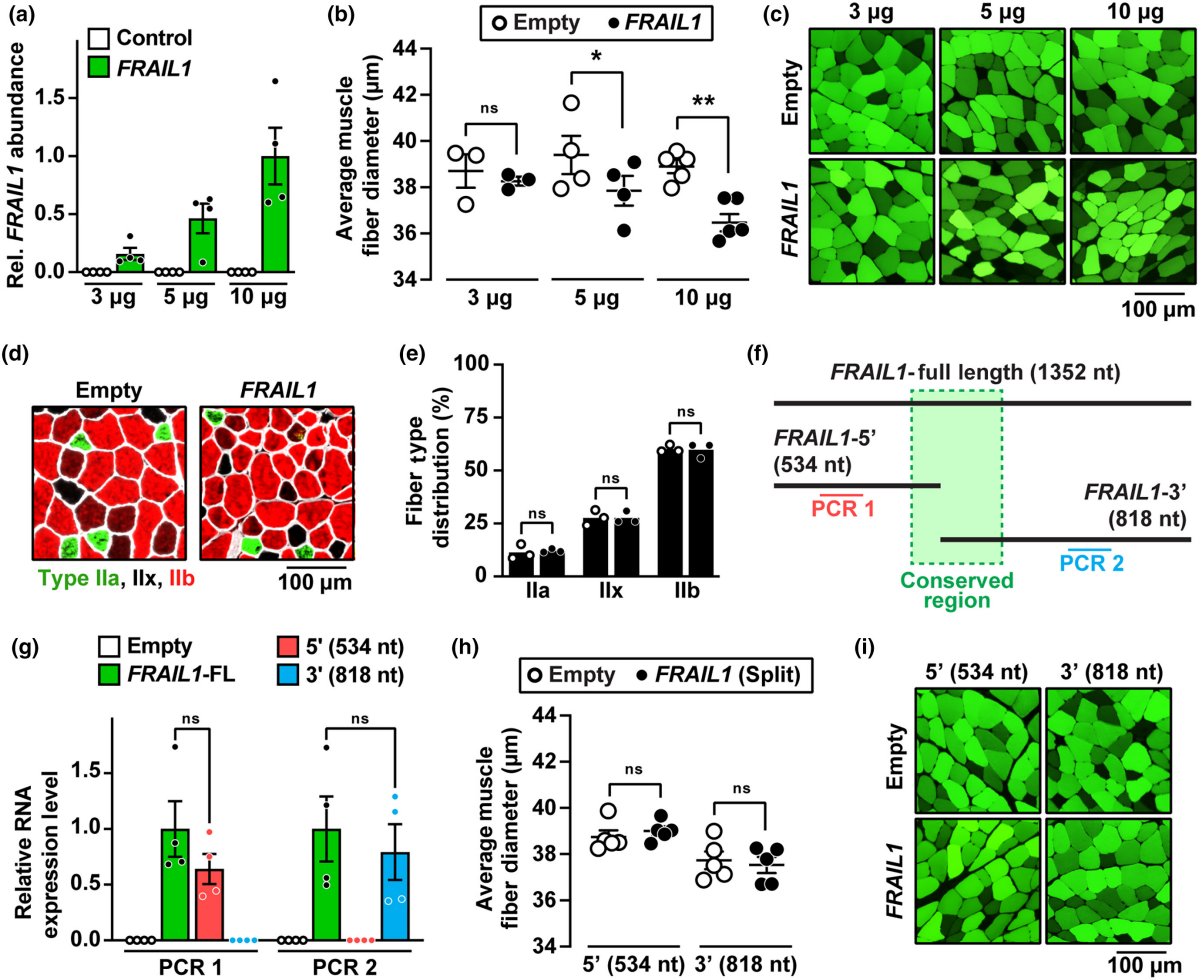
Forced expression of *FRAIL1* in mouse skeletal muscle causes muscle atrophy. (a–e) One TA muscle per mouse was transfected with empty plasmid (pcDNA3), and the contralateral TA in each mouse was transfected with plasmid encoding *FRAIL1* under the control of the cytomegalovirus (CMV) promoter, as indicated. Seven days posttransfection, bilateral TAs were harvested for RT‐qPCR analysis of *FRAIL1* expression (a) histological analysis of skeletal muscle fiber size (b, c) and immunofluorescence microscopy using antibodies targeting MYH2, MYH1, MYH4, and laminin for the quantification of muscle fiber type distributions (d, e). (f) Schematic diagram of the *FRAIL1* transcript and synthetic truncated isoforms. The region conserved between nonhuman primates is highlighted in green. (g–i) One TA muscle per mouse was transfected with 10 μg of empty plasmid, and the contralateral TA in each mouse was transfected with 10 μg of plasmid encoding one of the truncated forms of *FRAIL1* under the control of the CMV promoter, as indicated. Seven days posttransfection, bilateral TAs were harvested for RT‐qPCR analysis of RNA expression (g) and histological analysis of skeletal muscle fiber size (h, i). Shades of green (c, i) represent differential muscle fiber plasmid uptake following the transfection procedure. Horizontal bars indicate mean values from each group ±SEM, and *p* values were determined by paired (b, e, h) and unpaired (g) two‐tailed *t* tests. *p < 0.05, **p < 0.01.

The majority of human lncRNAs are poorly conserved across taxonomic phyla beyond primates, which represents a significant barrier to their experimental study (Johnsson et al., 2014; Kapusta & Feschotte, 2014; Pang et al., 2006; Washietl et al., 2014). Despite being among the most highly expressed lncRNAs in human skeletal muscle, *FRAIL1* is not conserved in rodents, and only ~20% of the *FRAIL1* nucleotide sequence is conserved between humans and nonhuman primates (Figure S4A). Nonetheless, we utilized mouse skeletal muscle as a model system to examine the effects of forced *FRAIL1* expression in mature skeletal muscle fibers. To this end, an in vivo plasmid‐based transfection of mouse tibialis anterior (TA) muscles with a plasmid encoding human *FRAIL1* was performed. The TA muscle was studied because its smaller size, unipennate architecture, and accessibility allow for more consistent transfection efficiency and reproducibility compared to other muscle groups such as the larger, multi‐pennate gastrocnemius muscle with variable distribution of fiber size. In contralateral TA muscles transfected with an empty control plasmid, *FRAIL1* lncRNA was not detected but was reliably expressed in *FRAIL1* plasmid transfected muscles (Figure 4a). Tibialis anterior muscle fibers expressing *FRAIL1* exhibited a dose‐dependent reduction in skeletal muscle fiber size compared to contralateral control muscles after 7 days. At the lowest plasmid concentration tested (3 μg/TA), there was no effect of forced *FRAIL1* expression on muscle fiber size; however, higher concentrations of plasmid (5 μg/TA and 10 μg/TA) induced a significant reduction in muscle fiber size (Figure 4b,c, Figure S4B). In mouse skeletal muscle, *FRAIL1*‐induced muscle atrophy was not accompanied by altered muscle fiber type composition, as evidenced by similar proportions of type IIa, IIx, and IIb fibers in all muscles transfected (Figure 4d,e). To test the hypothesis that the conserved region of *FRAIL1* plays a functional role in *FRAIL1*‐induced muscle atrophy, we generated plasmids expressing 5′ and 3′ truncated forms of *FRAIL1* (Figure 4f, Figure S4C–E). Following transfection in mouse TA muscles, both truncated forms were expressed at levels comparable to the full‐length *FRAIL1* transcript (Figure 4g), and neither construct altered muscle fiber size (Figure 4h,i, Figure S4F), indicating that this intact conserved region is necessary for *FRAIL1*‐induced muscle atrophy. These results demonstrate that ectopic overexpression of full‐length *FRAIL1* is sufficient to induce atrophy of mature mouse skeletal muscle fibers in vivo.

### 2.4 Forced *FRAIL1* expression alters the global skeletal muscle proteome and transcriptome

**FIGURE 5:**
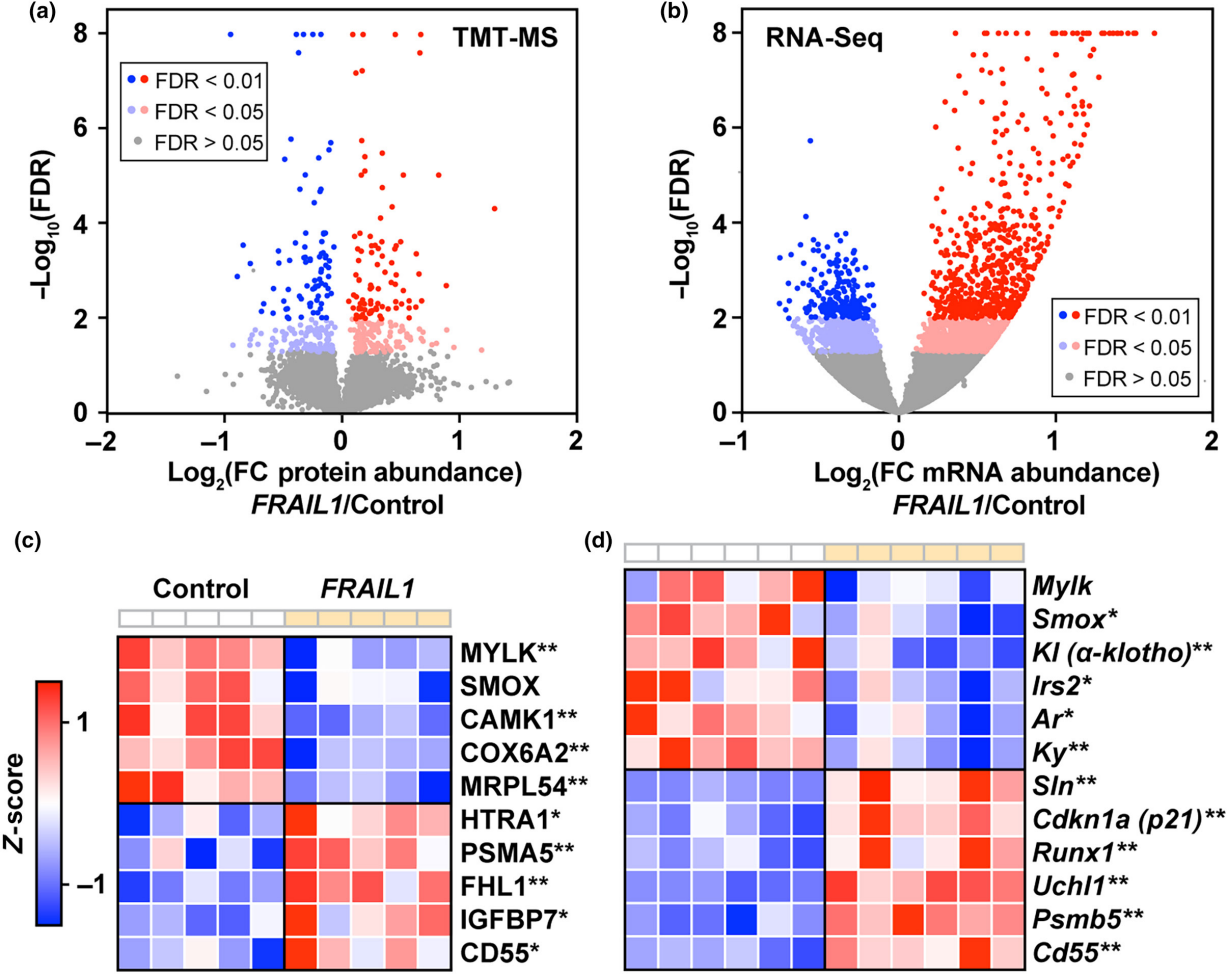
Forced *FRAIL1* expression alters the global skeletal muscle proteome and transcriptome. (a, b) One TA muscle per mouse (*n* = 5) was transfected with 10 μg of empty plasmid (pcDNA3), and the contralateral TA in each mouse was transfected with 10 μg of full‐length *FRAIL1* expressing plasmid. Seven days posttransfection, bilateral TAs were harvested and subjected to TMT‐mass spectrometry. In a separate cohort, one TA muscle per mouse (*n* = 6) was transfected with 10 μg of empty plasmid (pcDNA3), and the contralateral TA in each mouse was transfected with 10 μg of full‐length *FRAIL1* expressing plasmid. Seven days posttransfection, bilateral TAs were harvested and subjected to RNA‐seq analysis. (a, b) Volcano plots of differences in global protein (a) and transcript (b) abundance following ectopic *FRAIL1* expression. (c, d) Heatmaps of key skeletal muscle proteins (c) and mRNAs (d) differentially expressed in response to *FRAIL1* expression. False discovery rate (FDR) was determined by Benjamini‐Hochberg correction. * FDR ≤ 0.05, ** FDR ≤ 0.01.

To begin to understand the molecular changes underlying *FRAIL1*‐induced muscle fiber atrophy, we performed unbiased global proteomic and transcriptomic profiling of TA muscles ectopically expressing *FRAIL1* from two separate experimental cohorts of mice. This analysis revealed widespread changes in protein and gene expression that were more pronounced at the transcriptomic level compared to the proteomic level. A total of 5982 proteins were reliably quantified by tandem mass tag mass spectrometry (TMT‐MS) across experimental conditions with 199 (3.3%) increased and 178 (3.0%) decreased in abundance (FDR <0.05) with forced *FRAIL1* expression (Figure 5a, Table S2). By comparison, 15,538 RNA transcripts were reliably quantified by RNA‐Seq, of which 1238 (8.0%) were upregulated and 898 (5.8%) were downregulated (FDR <0.05) in response to *FRAIL1* (Figure 5b, Table S3). Consistent with our observations of equal muscle fiber type proportions by immunohistochemical staining (Figure 4d,e), forced *FRAIL1* expression did not alter the protein abundance of myosin heavy chain (MyHC) isoforms corresponding to the four major mouse skeletal muscle fiber types (MyHC‐Slow/MYH7, MyHC‐2A/MYH2, MyHC‐2X/MYH1, and MyHC2B/MYH4) (Table S2). *FRAIL1*‐regulated molecules included numerous proteins and mRNAs that have been implicated in the regulation of skeletal muscle mass and function. For example, *FRAIL1* expression reduced spermine oxidase (SMOX) protein and mRNA, an enzyme negatively associated with skeletal muscle atrophy across multiple diverse atrophy stimuli (Bongers et al., 2015; D'Ercole et al., 2022). Additional mRNA transcripts positively associated with skeletal muscle mass that were reduced by *FRAIL1* expression included Insulin Receptor Substrate 2 (*Irs2*) *(*Long et al., 2011 *)*, Androgen Receptor (*Ar*) (Hosoi et al., 2023), Kyphoscoliosis Peptidase (*Ky*) (Blanco et al., 2001; Hedberg‐Oldfors et al., 2016) and Klotho (*Kl*) (Arroyo et al., 2021; Clemens et al., 2021; Ohsawa et al., 2023). On the other hand, *FRAIL1* increased the expression of proteins and mRNAs negatively associated with skeletal muscle mass and quality. This included upregulation of proteases and proteasome subunits including increases at the protein level of Proteasome 20S Subunit Alpha 5 (PSMA5) and HtrA Serine Peptidase 1 (HTRA1) (Tiaden & Richards, 2013), as well as increased mRNA abundance of transcripts encoding Proteasome 20S Subunit Beta 5 (*Psmb5*) and Ubiquitin C‐Terminal Hydrolase L1 (*Uchl1*) (Gao et al., 2019; Reddy et al., 2018). Notably, forced *FRAIL1* expression also increased the abundance of mRNA encoding Sarcolipin (*Sln*), which is upregulated in immobilization‐induced muscle atrophy (Tomiya et al., 2019) and contributes to muscle wasting in models of muscular dystrophy (Niranjan et al., 2019; Schneider et al., 2013; Voit et al., 2017). Mouse skeletal muscles expressing *FRAIL1* also exhibited increases in several key molecules associated with signatures of cellular senescence. This included *FRAIL1*‐dependent increases in Cyclin Dependent Kinase Inhibitor 1A (*Cdkn1a/p21*), RUNX Family Transcription Factor 1 (*Runx1*), Insulin‐Like Growth Factor Binding Protein 7 (*Igfbp7*), and CD55 (Avelar et al., 2020; Englund et al., 2023; Saul et al., 2022; Zhang et al., 2022) (Figure 5c,d). These results indicate that *FRAIL1*‐mediated atrophy is associated with global proteomic and transcriptomic changes indicative of impaired skeletal muscle homeostasis and consistent with skeletal muscle atrophy.

### 2.5 Forced *FRAIL1* expression increases the abundance of proteins involved in RNA splicing and alters splicing of skeletal muscle mRNAs

**FIGURE 6:**
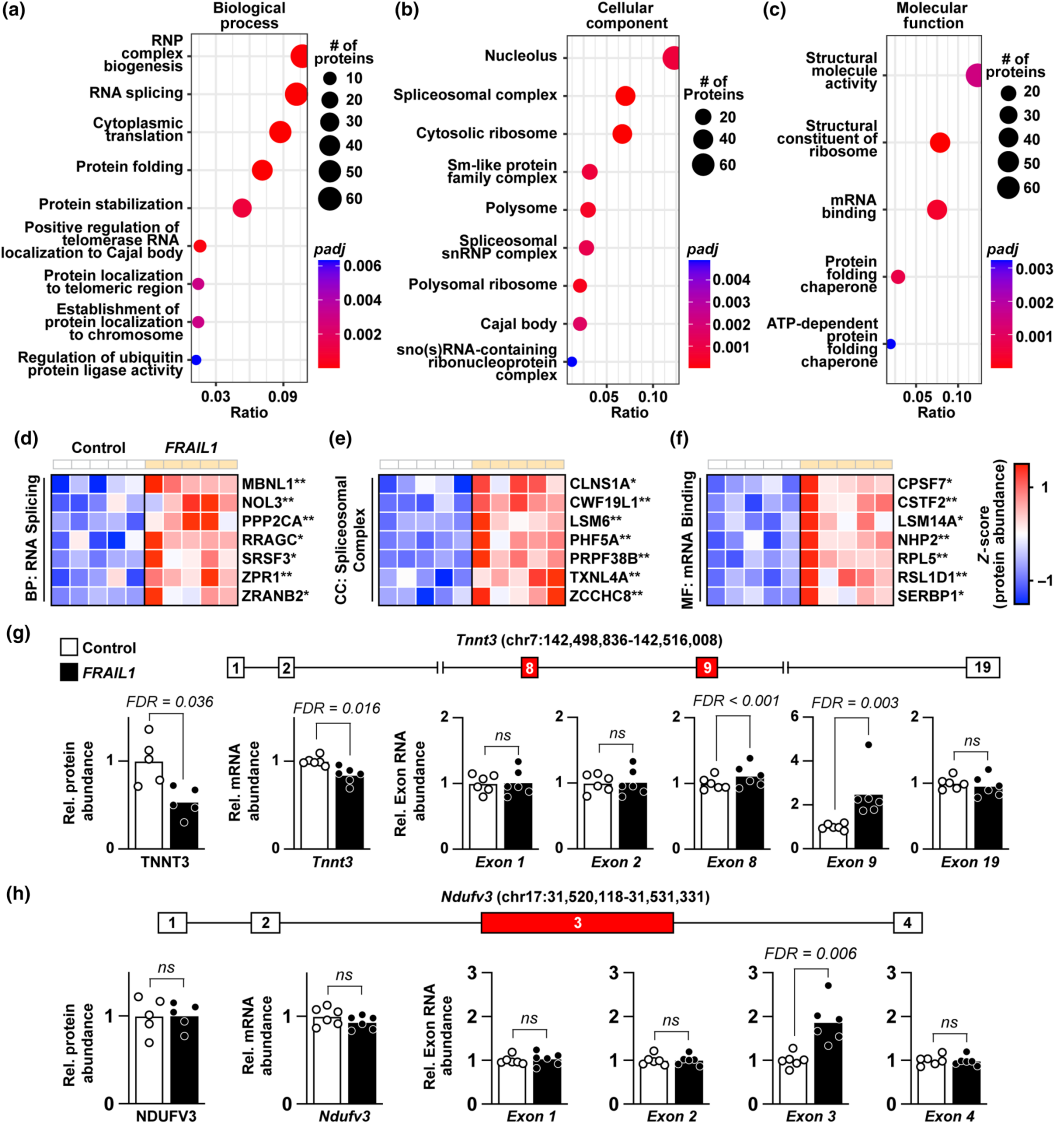
Forced *FRAIL1* expression increases the abundance of proteins involved in RNA splicing and alters the splicing of skeletal muscle mRNAs. (a–c) Enrichment plots of overrepresented GO terms among proteins increased by ectopic *FRAIL1* expression in mouse skeletal muscle for biological process (a), cellular component (b), and molecular function (c) categories. (d–f) Heatmaps of key upregulated proteins from the RNA splicing (d), spliceosomal complex (e), and mRNA‐binding (f) ontologies. (g) Diagram of the mouse *Tnnt3* locus and relative expression of TNNT3 protein, *Tnnt3* mRNA, and *Tnnt3* exons 1, 2, 8, 9, and 19 between control and *FRAIL1* expressing mouse skeletal muscles. (h) Diagram of the mouse *Ndufv3* locus and relative expression of NDUFV3 protein, *Ndufv3* mRNA, and *Ndufv3* exons 1–4 between control and *FRAIL1* expressing mouse skeletal muscles. False discovery rate (FDR) was determined by Benjamini‐Hochberg correction. *FDR ≤ 0.05, **FDR ≤ 0.01.

To identify molecular pathways that were dysregulated in response to *FRAIL1* expression in mouse skeletal muscle, we first performed gene ontology (GO) enrichment analysis of proteins that were increased in response to *FRAIL1* overexpression. The major theme emerging from this analysis was the upregulation of proteins involved in RNA splicing (Figure 6a), formation of the spliceosome (Figure 6b), and mRNA binding (Figure 6c). This enrichment of splicing‐associated pathways was driven by upregulation of proteins directly involved in mRNA processing including, but not limited to, Muscleblind Like Splicing Regulator 1 (MBNL1), Serine/Arginine Rich Splicing Factor 3 (SRSF3), Pre‐MRNA Processing Factor 38B (PRPF38B), and Cleavage/Polyadenylation Specific Factor 7 (CPSF7) (Figure 6d–f). Therefore, we hypothesized that forced *FRAIL1* expression induces differential splicing of mRNAs in skeletal muscle. To test this hypothesis, we performed an alternative splicing analysis of our transcriptomic data derived from mouse skeletal muscles expressing *FRAIL1* to identify differential exon usage normalized to changes in overall transcript abundance. This analysis identified 341 exons with differential abundance from a total of 240 unique genes (FDR < 0.1) (Table S4). Differentially spliced mRNAs included Troponin T3 (*Tnnt3*) and the mitochondrial accessory Complex I Subunit V3 (*Ndufv3*). Differential isoform expression of *Tnnt3* in skeletal muscle has been previously reported as a consequence of aging and limb immobilization, where it is associated with altered muscle contractile properties (Coble et al., 2015; Ravi et al., 2016; Sancisi et al., 2014; Schilder et al., 2011). In *FRAIL1* expressing mouse skeletal muscles, differential splicing of *Tnnt3* exons 8 and 9 was accompanied by decreased overall *Tnnt3* mRNA abundance and a reduction in TNNT3 protein (Figure 6g). By contrast, *FRAIL1* expression led to increased incorporation of *Ndufv3* exon 3 without altering total *Ndufv3* mRNA or NDUFV3 protein abundance (Figure 6h). The long isoform of NDUFV3, which incorporates a coding sequence contained within *Ndufv3* exon 3, is not typically expressed in skeletal muscle and is associated with differential complex I affinity to NADH (Dibley et al., 2017; Guerrero‐Castillo et al., 2017). Taken together, these results indicate that *FRAIL1* expression in skeletal muscle alters the abundance of proteins involved in RNA splicing and, either directly or indirectly, contributes to differential exon usage within mRNAs encoding protein isoforms with established functions in muscle.

### 2.6 Forced *FRAIL1* expression downregulates transcripts and proteins involved in the regulation of skeletal muscle structure, energy production, and metabolism

**FIGURE 7:**
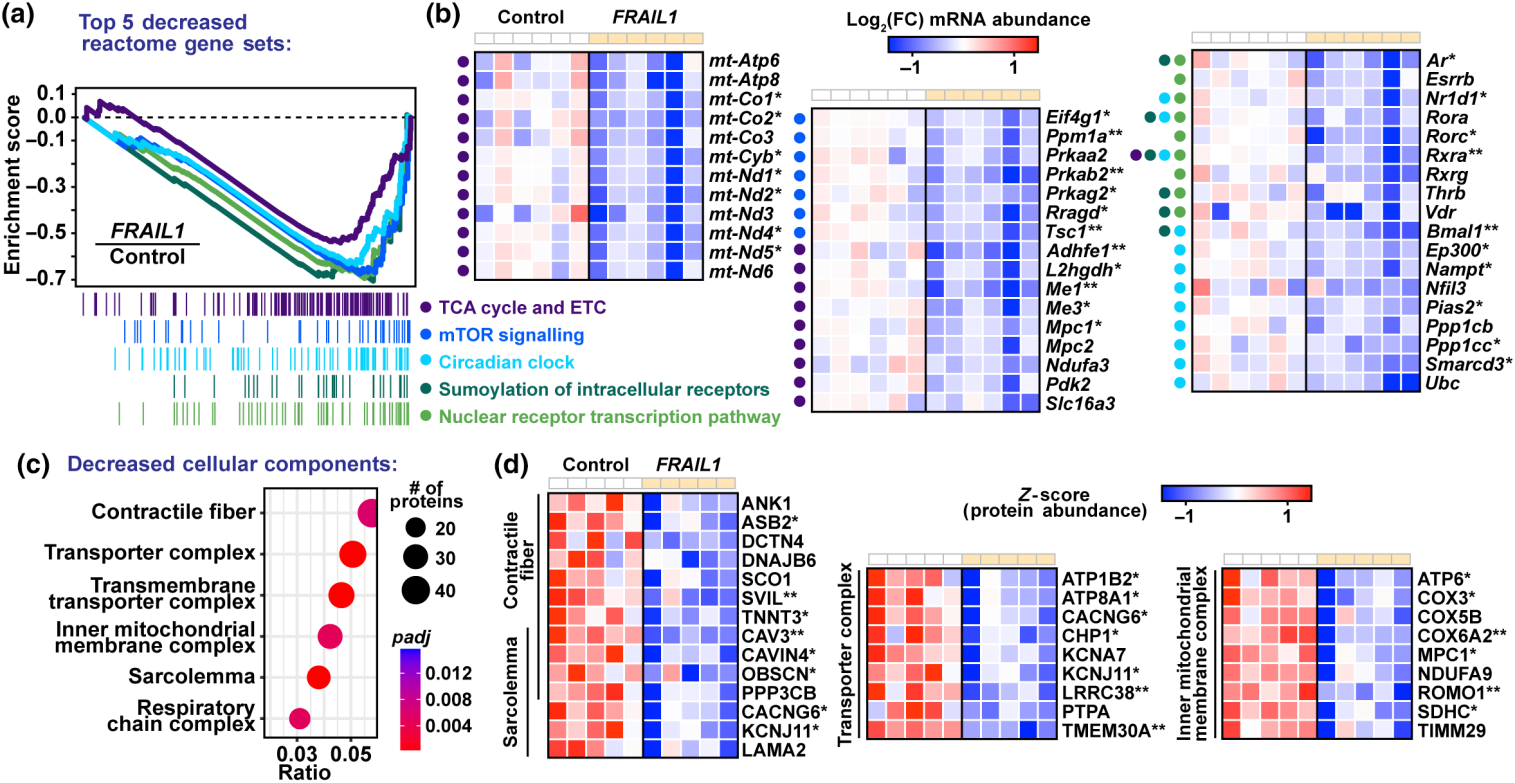
Forced *FRAIL1* expression downregulates transcripts and proteins involved in the regulation of skeletal muscle structure, energy production, and metabolism. Gene set enrichment analysis (GSEA) of RNA‐seq data was used to identify pathways that were repressed by *FRAIL1* expression in mouse skeletal muscle. (a, b) Enrichment plots of the top five Reactome gene sets that were repressed by *FRAIL1* expression in mouse tibialis anterior muscles (a) and heatmap of key individual transcripts from those gene sets (b) downregulated in response to *FRAIL1*. Colored dots left of heatmaps indicate the gene set(s) of origin for the associated gene. (c, d) Enrichment plots of overrepresented cellular component GO terms among skeletal muscle proteins decreased by ectopic *FRAIL1* expression (c) and key downregulated proteins from those ontologies (d). False discovery rate (FDR) was determined by Benjamini‐Hochberg correction. *FDR ≤ 0.05, **FDR ≤ 0.01.

To further characterize the effects of *FRAIL1* expression in mouse skeletal muscle, we performed gene set enrichment analysis (GSEA) of mRNAs that were dysregulated in response to *FRAIL1* expression. The dominant upregulated gene sets were involved in remodeling of the extracellular matrix and collagen turnover (Figure S5, Table S5). The top five most significantly repressed pathways have known roles in skeletal muscle homeostasis and were representative of overarching themes observed throughout downregulated pathways (Table S5). Specifically, *FRAIL1* expression reduced the expression of mRNAs involved in mitochondrial function, mTOR/AMPK signaling, and the circadian clock (Figure 7a). Reductions in the citric acid cycle and respiratory electron transport were driven by strong repression of transcripts from both mitochondrial and nuclear genomes encoding protein subunits of electron transport chain complexes and other mitochondrial enzymes (Figure 7b). The *FRAIL1*‐induced repression in mTOR/AMPK signaling gene sets was driven in part by decreased abundance of transcripts encoding multiple AMP‐activated protein kinase (AMPK) subunits (*Prkaa2*, *Prkab2*, *Prkag2)* (Figure 7b). AMPK is a key regulator of mTOR signaling, and normal skeletal muscle aging is associated with decreased levels of AMPK and increased activation of mTOR (Bujak et al., 2015; Reznick et al., 2007; Sandri et al., 2013). Last, *FRAIL1*‐induced reductions in the circadian clock and other nuclear receptor transcription pathways were driven by partially overlapping sets of mRNAs. Downregulated transcripts from these gene sets encode key transcription factors including Basic Helix–Loop–Helix ARNT Like 1 (*Bmal1*), Rev‐erb‐α (*Nr1d1*), and Retinoid X Receptor Alpha (*Rxra*) (Figure 7b).

Finally, we performed GO enrichment analysis on proteins reduced following forced *FRAIL1* expression in skeletal muscle. Decreased proteins were enriched for components of the contractile fiber and sarcolemma, as well as inner mitochondrial membrane and transporter complexes (Figure 7c). Muscles expressing *FRAIL1* exhibited decreased levels of the structural proteins Ankyrin 1 (ANK1), Obscurin (OBSC), and Supervillin (SVIL), three proteins that are critical for proper contractile function in striated muscle cells (Figure 7d; Armani et al., 2006; Giacomello et al., 2015; Hedberg‐Oldfors et al., 2020; Kontrogianni‐Konstantopoulos et al., 2003; Kontrogianni‐Konstantopoulos & Bloch, 2005). Furthermore, in accordance with decreased mitochondrial mRNA expression, *FRAIL1* expression depleted protein subunits of multiple electron transport chain complexes (Figure 7d). Taken together, these results indicate that *FRAIL1* expression in skeletal muscle is sufficient to decrease the abundance of mRNAs and proteins involved in key muscle metabolic signaling pathways, energy production and muscle structure.

## 3 DISCUSSION

In this study, we sought to characterize aging‐induced changes in human skeletal muscle lncRNA expression with the objective of identifying lncRNA molecules that might contribute to the salient morphological, biochemical, and functional features of aging skeletal muscle. Analysis of RNA‐Seq data obtained from human muscle biopsies from two independent cohorts of young and older individuals revealed *FRAIL1* as a highly abundant skeletal muscle‐enriched lncRNA that is robustly upregulated in older adults, particularly older females. Importantly, this understudied transcript was also recently highlighted as one of the top 10 skeletal muscle RNAs induced by aging (Tumasian III et al., 2021). *FRAIL1* is expressed within myonuclei and strongly enriched in skeletal muscle compared with other tissues, including cardiac muscle. We found that *FRAIL1* expression is sex‐dependent, and elevated *FRAIL1* levels coincide with decreased leg muscle strength and power. Furthermore, the *FRAIL1* promoter harbors binding sites for numerous skeletal muscle transcription factors whose mRNA expression levels mirror the pattern of *FRAIL1* expression across sex and age groups.

Previous studies investigating the roles of lncRNAs in skeletal muscle have primarily focused on lncRNAs that are conserved in nucleotide sequence between humans and rodents (Neppl et al., 2017; Wohlwend et al., 2021). However, like the majority of lncRNAs, only a portion of the *FRAIL1* primary nucleotide sequence (~20%) is conserved between humans and primates (Johnsson et al., 2014; Kapusta & Feschotte, 2014; Pang et al., 2006; Washietl et al., 2014). Despite this, to study the cellular effects of *FRAIL1* expression in mature skeletal muscle fibers, we utilized plasmid‐based transfection of the mouse tibialis anterior. This method enables the controlled in vivo expression of transgenes within terminally differentiated myofibers (Hughes et al., 2022). In this model, forced *FRAIL1* expression is sufficient to induce muscle fiber atrophy, and this effect is dependent on the intact region of *FRAIL1* that is conserved between humans and primates. Nevertheless, the lack of endogenous *FRAIL1* expression in mouse skeletal muscle prevents the loss of function experiments that would provide clues into its normal function. At this early stage, we have insufficient information to speculate on the normal function of *FRAIL1* except that we cannot ignore the possibility that the function of *FRAIL1* is more nuanced than solely driving muscle atrophy and it may be part of a species‐specific muscle regulatory process.

To begin to understand the cellular effects of forced *FRAIL1* expression, we performed unbiased transcriptomic and proteomic profiling of mouse skeletal muscle expressing *FRAIL1*. This analysis revealed changes in mRNA and protein abundance consistent with skeletal muscle atrophy. These changes included increased expression of *Cdkn1a/p21* and other senescence‐associated mRNAs (Avelar et al., 2020; Englund et al., 2023; Fox et al., 2014; Saul et al., 2022; Zhang et al., 2022), reduced expression of spermine oxidase mRNA and protein (Bongers et al., 2015; D'Ercole et al., 2022), repression of mitochondrial pathways at the mRNA and protein levels (Joseph et al., 2012; Lanza & Nair, 2009; Petersen et al., 2003; Short et al., 2005; Trounce et al., 1989) and decreased abundance of key structural components of the contractile fiber and sarcolemma (Armani et al., 2006; Giacomello et al., 2015; Hedberg‐Oldfors et al., 2020; Kontrogianni‐Konstantopoulos et al., 2003; Kontrogianni‐Konstantopoulos & Bloch, 2005). These results not only implicate *FRAIL1* in the pathogenesis of age‐related muscle atrophy but also highlight the potential use of model organisms in the study of lncRNA biology, regardless of primary nucleotide sequence conservation (Ghanam et al., 2022).

Our study has several limitations that should be considered when interpreting the implications of our results. First, the current study utilized the human vastus lateralis as a readout of the aging muscle transcriptome, and the mouse tibialis anterior as an experimental model system for the effects of forced *FRAIL1* expression in muscle fibers. Although the muscle atrophy evident with forced *FRAIL1* expression did not appear to be fiber type dependent, it will nevertheless be important to interrogate the expression of *FRAIL1*, and the consequences of ectopic *FRAIL1* expression, in other muscle groups with distinct anatomical locations, phenotypes, and fiber type compositions. Second, the functional characterization of the effects of *FRAIL1* presented in this study is limited to one species, mice, wherein the primary *FRAIL1* nucleotide sequence is not conserved. Although we believe that this represents an appropriate model system to interrogate the effects of *FRAIL1* expression in mature myofibers, we also acknowledge that further studies in other species are necessary to confirm the role of *FRAIL1* in age‐related muscle atrophy. Third, this study represents an initial preliminary investigation of *FRAIL1* in a single muscle group, and follow‐up experiments in additional muscle groups and additional biological replicates are essential. Last, the transcriptomic and proteomic data presented in this study are correlative and do not directly implicate *FRAIL1* in the observed muscle atrophy‐related changes. In the future, it will be important to characterize the effects of *FRAIL1* depletion within human skeletal muscle cells, as well as to perform mechanistic investigations such as identifying *FRAIL1*‐interacting proteins within skeletal muscle fibers.

In summary, we identify *FRAIL1* as among the most highly abundant aging‐induced human skeletal muscle‐enriched lncRNAs, whose forced expression is sufficient to induce myofiber atrophy and recapitulate molecular signatures evident in human skeletal muscle with aging. Although this study represents an initial step in investigating the potential role of *FRAIL1* in age‐related skeletal muscle dysfunction, further characterization of its molecular function and interaction with other cellular signaling pathways that influence muscle mass and function with aging will be important before its potential as a therapeutic target or clinical marker for the prevention or monitoring of age‐related skeletal muscle atrophy can be evaluated.

## 4 MATERIALS AND METHODS

### 4.1 Human protocols

The human data came from two different cohorts of young and older males and females. The initial discovery cohort (Figure 1) consisted of young (*n* = 22; 25 ± 5 years) and older (*n* = 14; 71 ± 5 years) adults from previously published studies (Hart et al., 2019; Robinson et al., 2017). The validation cohort for Figure 2 consisted of a larger number of young (*n* = 30; 27.0 ± 4.11 years) and older (*n* = 50; 71.4 ± 4.7 years) participants that have been described previously (Kunz et al., 2022; Zhang et al., 2021). All participants provided informed written consent, and studies were approved by the Mayo Clinic Institutional Review Board and conducted in accordance with the Declaration of Helsinki. Potential participants were excluded if they engaged in structured exercise training (>20 min, twice weekly) or if they exhibited signs of cardiovascular disease, diabetes, untreated thyroid disorders, renal disease, blood clotting disorders, or smoking. Further, participants were excluded if they were taking insulin, insulin sensitizers, corticosteroids, sulfonylureas, barbiturates, peroxisome proliferator‐activated receptor γ agonists, β blockers, opiates, and tricyclic antidepressants. Following the screening, participants completed study visits involving body composition evaluation by dual‐energy X‐ray absorptiometry (DEXA; GE Lunar iDXA, GE Healthcare, Chicago, IL, USA). Muscle strength was determined from measurements of unilateral 1‐repetition maximum (1‐RM) during knee extension (Keiser Air300, Keiser Corporation, Fresno, CA, USA). Following habituation and light warm‐up, participants performed a series of single‐repetition attempts at increasing workloads, each separated by 3 min of rest, until the maximum load that could be moved through the entire range of motion was reached (Kunz et al., 2022). At least 7 days later, participants reported for a muscle biopsy of the *vastus lateralis* muscle. The biopsy was collected in an inpatient setting following 3 days of standardized meals (20% protein, 50% carbohydrates, and 30% fat) to maintain body weight based on caloric requirement using Harris‐Benedict equations. On the evening of inpatient hospital admission, a light snack was provided at 2100 then participants remained fasting overnight. The following morning at 0700, biopsies (~200 mg) were collected from the *vastus lateralis* with analgesia by 2% lidocaine with sodium bicarbonate buffer.

### 4.2 LncRNA profiling from human tissue

Total RNA was isolated from ~20 mg of biopsy sample using a commercially available kit according to manufacturer's instructions (RNeasy Fibrous Tissue, Qiagen). Total RNA was eluted in 100 μL of PCR grade water and concentration was adjusted to 50 ng/μL using a spectrophotometer (Nanodrop, Thermo Scientific, Waltham, MA). RNA libraries were prepared for sequencing using the TruSeq RNA Sample Prep Kit v2 (Illumina, San Diego, CA), following the manufacturer's recommendations. Briefly, poly‐A mRNA was purified from 100 ng total RNA using oligo dT magnetic beads. Purified mRNA was fragmented at 95°C for 8 min and eluted from the beads. Double‐stranded cDNA was made using SuperScript III reverse transcriptase, random primers (Invitrogen, Carlsbad, CA) and DNA polymerase I and RNase H. The cDNA ends were repaired, and an “A” base was added to the 3′ ends. TruSeq paired end index DNA adaptors (Illumina, San Diego, CA) with a single “T” base overhang at the 3′ end were ligated and the resulting constructs were purified using Agencourt AMPure SPRI beads (Beckman Coulter, Chaska, MN). Adapter‐modified DNA fragments were enriched by 12 cycles of PCR using TruSeq PCR primers (Illumina, San Diego, CA). The concentration and size distribution of the libraries were determined on an Agilent Bioanalyzer DNA 1000 chip and Qubit fluorometry (Invitrogen, Carlsbad, CA). Samples were divided into batches of eight and their indexed libraries were pooled at equimolar concentrations. A pooled library was loaded onto paired‐end flow cells at a concentration of 8.5 pM to generate cluster densities of 700,000/mm^2^, following the standard protocol for the Illumina cBot and cBot paired‐end cluster kit version 3. The flow cells were sequenced as 51X2 paired‐end reads on a HiSeq 2000 sequencer (Illumina, San Diego, CA) using TruSeq SBS sequencing kit version 3 (Illumina, San Diego, CA) and HCS version 2.0.12.0 data collection software. Base calling is performed using Illumina's RTA version 1.17.21.3. On average, 56 million reads were generated for each sample. The RNA‐Seq data were analyzed using the MAPRSeq (version 1.2.1) system for RNA‐Sequencing data analysis.

### 4.3 Mouse protocols

Animals were housed in colony cages at 21°C with 12‐h light/12‐h dark cycles and with ad libitum access to standard chow (PicoLab Rodent 5053) and water throughout the study. For all mice used in the study, male C57BL/6N mice were obtained from Charles River Laboratory between 6 and 8 weeks of age and were used for experiments within 2 weeks of their arrival. All animal procedures were approved by the Institutional Animal Care and Use Committee of The Mayo Clinic.

### 4.4 Mouse skeletal muscle plasmid transfection

Plasmids expressing full‐length and truncated forms of *FRAIL1* were generated by cloning *FRAIL1* cDNA into pcDNA3.1(+) while un‐modified pcDNA3.1(+) was used as an empty control plasmid in all transfection experiments. In experiments analyzing skeletal muscle fiber size, all muscles were co‐transfected with 5 μg of plasmid encoding eGFP under the control of the CMV promoter. Tibialis anterior (TA) muscles were transfected via electroporation as described previously (Ebert et al., 2010). Briefly, mice were anesthetized, with 91 mg/kg ketamine and 9.1 mg/kg xylazine; hind limbs were shaved; and the tibialis anterior muscles (TAs) were injected with 30 μL of 0.4 units/μL bovine placental hyaluronidase (Sigma) resuspended in sterile saline. One hour later, mice were re‐anesthetized. The TAs were then injected with plasmid DNA in sterile saline, coated with ultrasound jelly, and subjected to three 50‐ms pulses of 250 V/cm using an ECM‐830 electroporator. Following transfection, mice were returned to their cages to resume normal activities for 7 days before muscle harvest.

### 4.5 Mouse skeletal muscle histological analyses

For analysis of muscle fiber size, harvested TA muscles were immediately fixed in 4% paraformaldehyde for 16 h at 4°C and then incubated in 30% sucrose for 16 h. The muscles were then embedded in optimal cutting temperature (O.C.T.) compound (Tissue‐Tek, catalog no. 4583), snap frozen, and a Cryostar HM525 NX (Epredia) was used to collect 10‐μm sections from muscle mid‐bellies. For analysis of muscle fiber types, harvested TA muscles were immediately placed in O.C.T. compound, snap frozen, and 10‐μm sections were collected as described above. For immunofluorescence staining, cryosections were immersed in acetone for 10 min at −20°C, rinsed three times with PBS (Corning, product no. 21‐040‐CV), blocked in Buffer A (PBS, pH 7.4, containing 0.5% Triton X‐100 and 5% horse serum) for 20 min at 21°C, rinsed three times with PBS, incubated for 1 h at 21°C in Buffer A supplemented with primary antibodies, rinsed three times with PBS, and incubated for 1 h at 21°C in Buffer A supplemented with the appropriate secondary antibodies, rinsed three times with PBS, and then mounted in ProLong Gold Antifade reagent (Invitrogen, catalog no. P36930) and overlayed with a coverslip (FisherScientific, catalog no. 12‐545k). Primary and secondary antibody‐containing solutions were composed as follows: a 1:250 dilution of rabbit polyclonal anti‐laminin IgG (Sigma, catalog no. L9393) and a 1:500 dilution of Alexa Fluor 568‐conjugated anti‐rabbit‐IgG (Invitrogen, catalog no. 11011) were used for laminin staining; a 1:10 dilution of mouse monoclonal anti‐Myh4 IgM (DSHB, BF‐F3) and a 1:500 dilution of Alexa Fluor 647‐conjugated anti‐mouse IgM (Invitrogen, catalog no. A21238) were used for Myh4 (type IIb) staining; a 1:25 dilution of mouse monoclonal anti‐Myh2 IgG1 (DSHB, SC‐71) and a 1:500 dilution of Alexa Fluor 488‐conjugated anti‐mouse IgG1 (Invitrogen, catalog no. A21121) were used for Myh2 (type IIa) staining; and a 1:25 dilution of mouse monoclonal anti‐Myh7 IgG2b (DSHB, BA‐F8) and a 1:500 dilution of Alexa Fluor 350‐conjugated anti‐mouse IgG2b (Invitrogen, catalog no. A21140) were used for Myh7 (type I) staining. Muscle fibers that did not stain positive for any of the three stained Myh isoforms were assigned to the unstained Myh isoform Myh1 (type IIx). All sections were examined and photographed using a Nikon Eclipse Ti2‐U inverted microscope equipped with Nikon Elements Ar software package and DAPI (catalog no. 96370), GFP (catalog no. 96732), TRITC (catalog no. 96374), and Cy5 (catalog no. 96376) filter cubes from IDEX Health and Science (Rochester, NY). Image analysis was performed using the Nikon Elements General Analysis (GA3) software using the “Object Count” function. Skeletal muscle fiber size was analyzed by measuring the lesser diameter (minimal Feret diameter) and cross‐sectional area (CSA) of muscle fibers. The minimal Feret diameter is less susceptible to errors related to the orientation of sectioning angles (oblique) that may distort actual cross‐sectional area values.

### 4.6 Mouse skeletal muscle RT‐qPCR

Freshly excised mouse TA muscles were immediately frozen in liquid *N*_2_ and stored at −80°C. Muscle RNA was extracted using TRIzol solution (Thermofisher, catalog no. 15596018) and then homogenized with three 30 s cycles of Precellys Evolution (Bertin Technologies, catalog no. PE000062) homogenizer set at 8200 rpm. RNA was then extracted following the manufacturer's directions and purified using the Turbo DNA‐free kit (Invitrogen, catalog no. AM1907). Reverse transcription of skeletal muscle RNA was performed using a high‐capacity cDNA reverse transcription kit (Applied Biosystems, catalog no. 4368814). RT‐qPCR analyses were performed with a QuantStudio 6 Pro Real‐Time PCR System (ThermoFisher, catalog no. A43054) using *Power*SYBR Green PCR Master Mix (Applied Biosystems, Ref. 4367659). All qPCR samples were run in triplicate, and the cycle threshold (Ct) values were averaged. For data analysis, the ΔΔ*C*_t_ method was utilized, with 36B4 mRNA serving as the invariant control. Primers used, as indicated in Figure 3f, were PCR1‐Forward (TGAGCTCTCACTGGCAAACTAT), PCR1‐Reverse (CTTCATACAGTTGAAAGAGGCAC), PCR2‐Forward (GCCAGCATTACCCTGATACC), and PCR2‐Reverse (CACTCGATCATGGTGAATGCT).

### 4.7 Mouse skeletal muscle RNA‐seq

Freshly excised mouse TA muscles were immediately frozen in liquid N_2_ and stored at −80°C. Muscle RNA was extracted using TRIzol solution (Thermofisher, catalog no. 15596018) and then homogenized with three 30 s cycles of Precellys Evolution (Bertin Technologies, catalog no. PE000062) homogenizer set at 8200 rpm. RNA was then extracted following the manufacturer's directions and purified using the RNeasy kit and RNase‐free DNase Set (Qiagen) according to the manufacturer's protocol. Total RNA was submitted to GENEWIZ standard RNA‐seq service (USA; www.GENEWIZ.com) that provided a standard Illumina mRNA library and generated 150 bp pair‐end reads on an Illumina NovaSeq (Illumina) Sequencing reads were trimmed to remove adaptor sequences using Trimmomatic (Bolger et al., 2014) and mapped to the *M. musculus* reference genome mm10 using RNA‐star (v2.7.8a) (Dobin et al., 2013). FeatureCounts (v2.0.1) was used to count the number of reads uniquely mapping to annotated genes (Liao et al., 2014) and was used for normalization and differential gene expression analysis using DESeq2 (v2.11.40.7) (Love et al., 2014) on the online platform Galaxy (Afgan et al., 2018). Transcripts were then preranked (log_2_FC*–log_10_(*p*‐value)) and subjected to Gene Set Enrichment Analysis (GSEA; Subramanian et al., 2005) with the following parameters: classic scoring scheme, meandiv normalization, 10,000 permutations, Mouse Gene Symbol Remapping to Human Orthologs chip platform, and the Reactome v.2022.1 gene sets database. Alternative splicing analysis was performed using the Bioconductor package DEXSeq (v1.44.0) to quantify differential exon usage (Anders et al., 2012).

### 4.8 Mouse skeletal muscle proteomic sample preparation

Freshly excised mouse TA muscles were immediately frozen in liquid N_2_ and stored at −80°C. Whole, frozen TA muscles were then pulverized to powder using a stainless‐steel homogenizing apparatus embedded in dry ice. Five biological replicates from each group were pulverized, and then powdered tissue was weighed, lysed at 21°C for 3 s in 20× (v/w) Buffer B (50 mM Tris, pH 8.0, 150 mM sodium chloride, 10% SDS, 1% NP‐40, 0.5% sodium deoxycholate, 1 mM EDTA, complete mini‐EDTA‐free protease inhibitor, and PhosStop phosphatase inhibitor) using a handheld Pellet Pestle Motor (Kontes), and immediately frozen. Samples were then thawed, centrifuged at 20,000×*g* for 5 min at 21°C, and clarified supernatant was saved in a new tube. Soluble protein concentration was measured using the Pierce BCA Protein Assay (ThermoFisher, catalog no. 23225), and 200 μg of soluble protein from each sample was digested with trypsin/Lys‐C mixture (Promega, catalog no. V5072) at 37°C for 16 h, using S‐Trap Mini columns from Protifi, per manufacturer's instructions. Resultant peptide isolate concentrations were measured with a quantitative colorimetric peptide assay (Pierce, catalog no. 23275). An aliquot of 100 μg peptides from each replicate sample was dried down in a SpeedVac, reconstituted in 100 mM TEAB (ThermoScientific, catalog no. 90114), and labeled with tandem mass tags (TMT 10‐plex) according to manufacturer's specifications (ThermoScientific, catalog no. 90406). Following TMT labeling, equal sample mixing, C18 clean‐up, and lyophilization, samples were suspended in 10 mM ammonium formate, pH 9.0, and fractionated via high pH RP‐HPLC (Agilent). Peptide separation and fraction was done using a 4.6 × 150 mm × 3.5 μm XBridge C18 column (Waters) over a 90 min gradient from 2% to 40% mobile phase B. Mobile phase A was 10 mM ammonium formate, and mobile phase B was 10 mM ammonium formate in 80% acetonitrile. A total of 96 fractions were collected and subsequently concatenated to 24 fractions. Approximately 3 μg from each fraction was transferred to a sample vial, dried in a SpeedVac, suspended in 0.2% formic acid/0.1% trifluoroacetic acid/0.002% zwittergent 3–16 sample buffer, and subjected to mass spectrometry.

### 4.9 Mass spectrometry

TMT‐labeled peptide from each sample was analyzed by nanoflow liquid chromatography electrospray tandem mass spectrometry using a Thermo Ultimate 3000 RSLCnano HPLC autosampler system coupled to an Orbitrap Fusion Lumos mass spectrometer (Thermo Scientific). Peptides (500 ng) were loaded on a 33 μL EXP stem trap packed with HALO C18 resin (2.7 μM, 90 Å, Optimize Technologies). Peptides were eluted at a flow rate of 350 ηL/min from the trap through a 100 μm × 30 cm PicoFrit column (New Objective) packed in‐house with Acclaim Pepmap C18 resin (Thermo Fisher). Chromatography was performed using a 2% to 35% gradient of Solvent B over 120 min (Solvent A: 98% water/2% acetonitrile/0.2% formic acid, Solvent B: 80% acetonitrile/10% isopropanol/10% water/0.2% formic acid). The Fusion Lumos mass spectrometer was set to acquire ms1 survey scans from 340 to 1600 *m*/*z* at a resolution of 120,000 (at 200 *m/z*) with the automatic gain target (AGC) set at 4e5 ions and a maximum ion inject time of 50 ms. Survey scans were followed by ms2 high energy collision dissociation (HCD) set at 38%, resolution of 50,000 (at 200 *m*/*z*), an AGC target of 1e5 ions, a maximum ion inject time of 120 ms, and the isolation window set at 0.07 Da. Dynamic exclusion placed selected ions on an exclusion list for 60 s.

### 4.10 Proteomic data analysis

Proteome Discoverer v3.0 software was used for database searching and extracting quantitative values for downstream analysis. Mass spectrometry files were searched using the Sequest search engine against a mouse‐specific, Swiss‐Prot FASTA database (https://www.uniprot.org, Taxon ID: 10090, Uniprot release 2021_03). Search settings included precursor mass tolerance of 10 ppm, fragment mass tolerance of 0.02 Da, variable modification oxidation methionine, and static modifications for carbamidomethyl cysteine and TMT 6‐plex labels on lysines and N‐termini. Enzyme specificity was set to trypsin, and a maximum of two missed cleavages were allowed. A false discovery rate (FDR) of 1% was required for both proteins and peptides, using a target‐decoy approach. Protein data were exported from the Proteome Discoverer result file (*.pdResult) and differential quantification was calculated from reported TMT protein intensities using a generalized linear model in R (Ayers‐Ringler et al., 2016). Briefly, log_2_‐transformed and quantile‐normalized intensities, observed in control and *FRAIL1* samples, were modeled using a Gaussian‐linked generalized linear model. An ANOVA test was used to detect differentially expressed proteins between experimental conditions, and the resulting p‐values were FDR‐corrected using the Benjamini‐Hochberg procedure. Gene ontology (GO) enrichment analysis of differential protein expression was performed using clusterProfiler (v4.6.0) and the enrichplot package to identify enriched GO terms (*q*‐value cutoff 0.05) in up‐ or downregulated proteins (FDR < 0.2) (Wu et al., 2021). GO terms were simplified using the simplify function of clusterProfiler.

### 4.11 Statistics

Unless noted separately in the Materials and Methods, all statistical analyses were performed with GraphPad Prism using statistical tests described in figure legends.

## AUTHOR CONTRIBUTIONS

Ian Lanza, Kevin Gries, Hawley Kunz, and Matthew Miller designed the studies, initiated the project, collected the samples, and wrote the manuscript. George Marcotte, Zachary Ryan, Scott Ebert, and Bonnie Arendt coordinated and performed the experiments. Chris Adams provided conceptual inputs for the project. Surendra Dasari and Matthew Strub provided bioinformatic analyses.

## FUNDING INFORMATION

Funding for this work came from NIA R01 AG054454, NIA R01 AG060637, NIAMS R01 AR071762, NIAMS R44 AR069400, and NIA R44 AG047684.

## CONFLICT OF INTEREST STATEMENT

The authors declare that they have no known competing financial interests or personal relationships that could influence this work.

## Supporting information


Figure S1.

Figure S2.

Figure S3.

Figure S4.

Figure S5.

Table S1.

Table S2.

Table S3.

Table S4.

Table S5.

## Data Availability

RNA‐sequencing and proteomics data are available from the corresponding author upon reasonable request. The RNA‐seq data are available in the gene expression omnibus (GEO) accession number: GSE249356.
