# Supplementary figures and images for: Human myofiber‐enriched aging‐induced lncRNA FRAIL1 promotes loss of skeletal muscle function
Source: Aging Cell. 2024 Jan 31;23(4):e14097. doi: 10.1111/acel.14097 (PMC11019130; doi:10.1111/acel.14097)

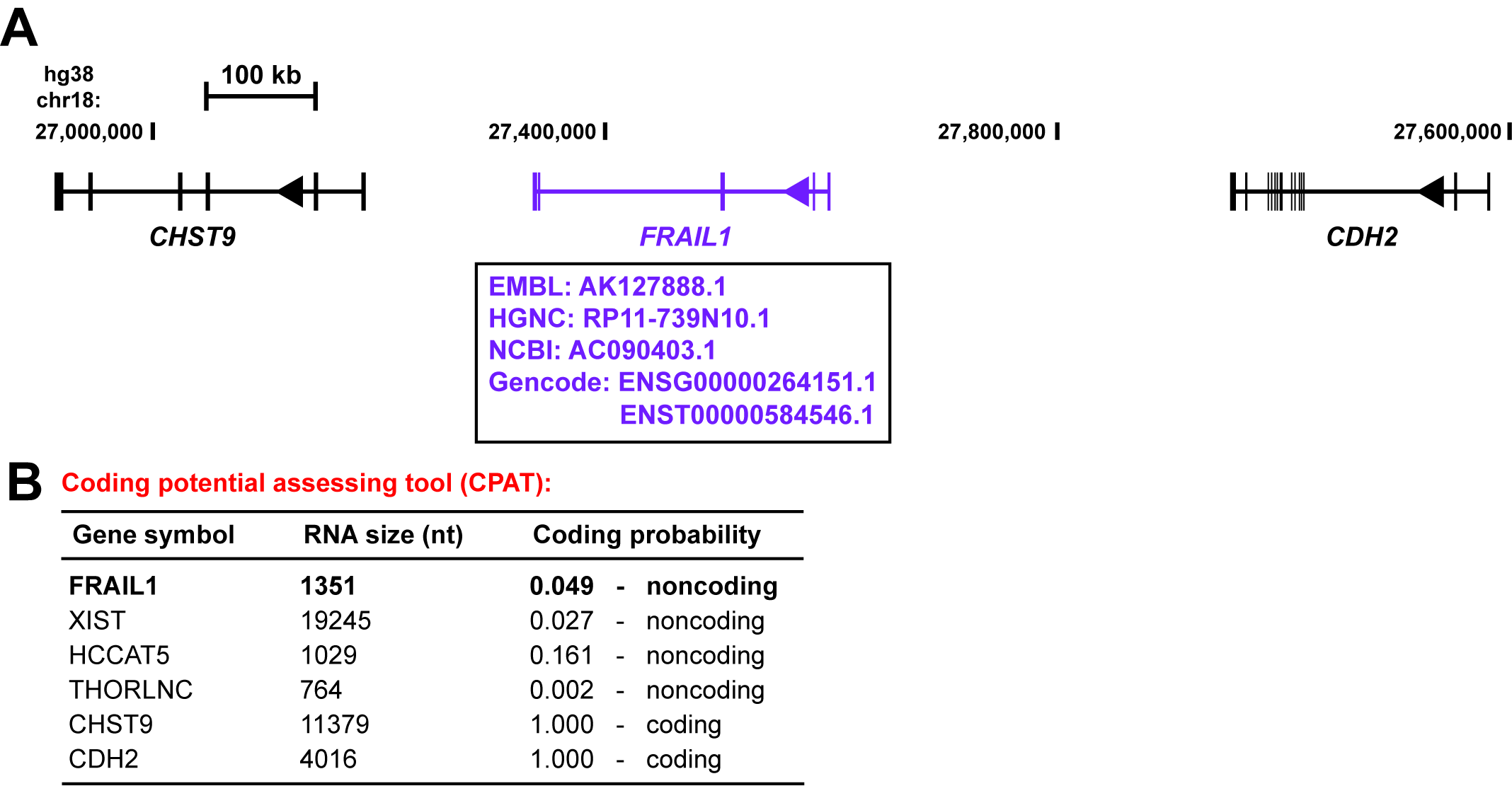

Supplement: Supplementary file 1 — Figure S1. [file ACEL-23-e14097-s006.tif]

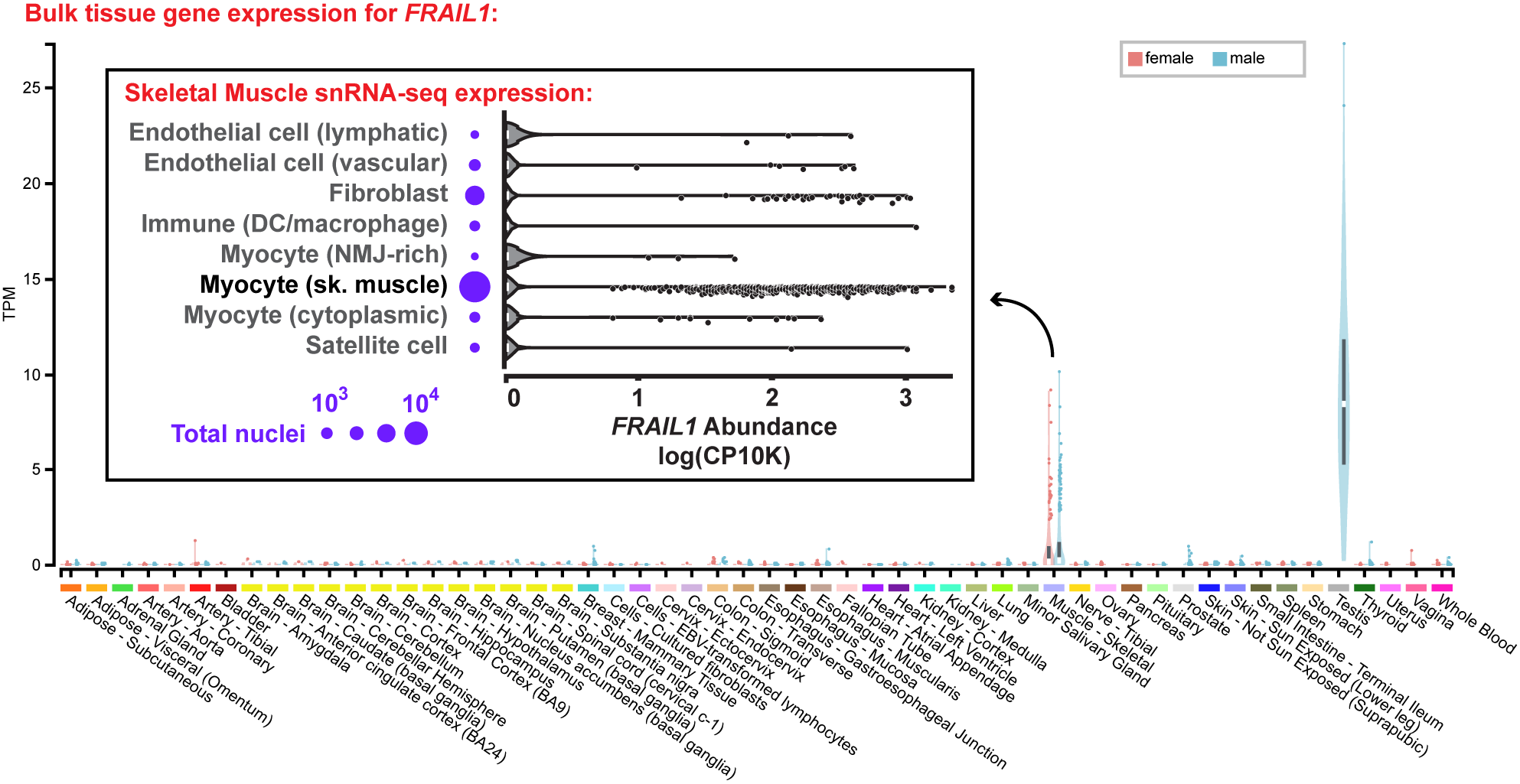

Supplement: Supplementary file 2 — Figure S2. [file ACEL-23-e14097-s007.tif]

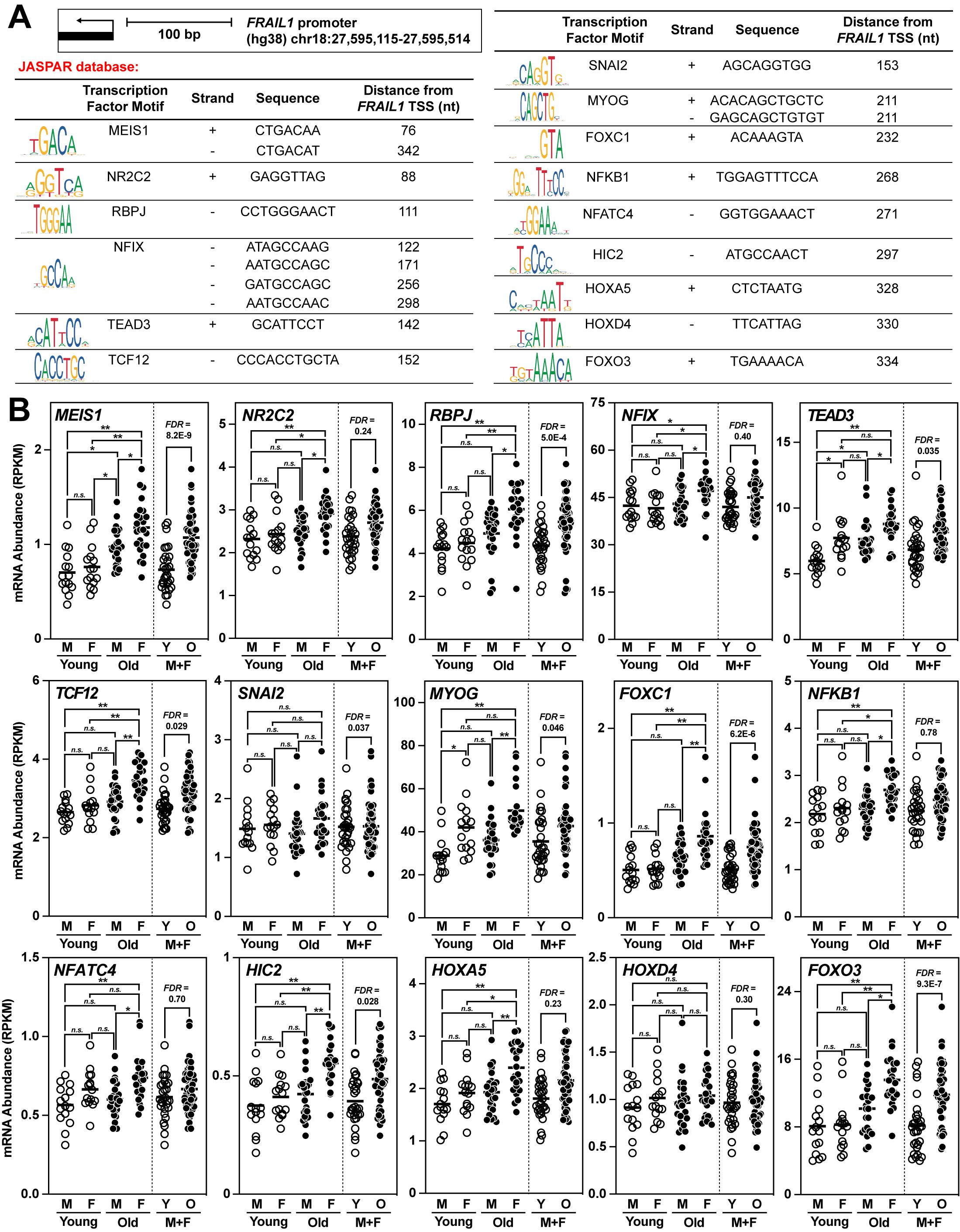

Supplement: Supplementary file 3 — Figure S3. [file ACEL-23-e14097-s009.tif]

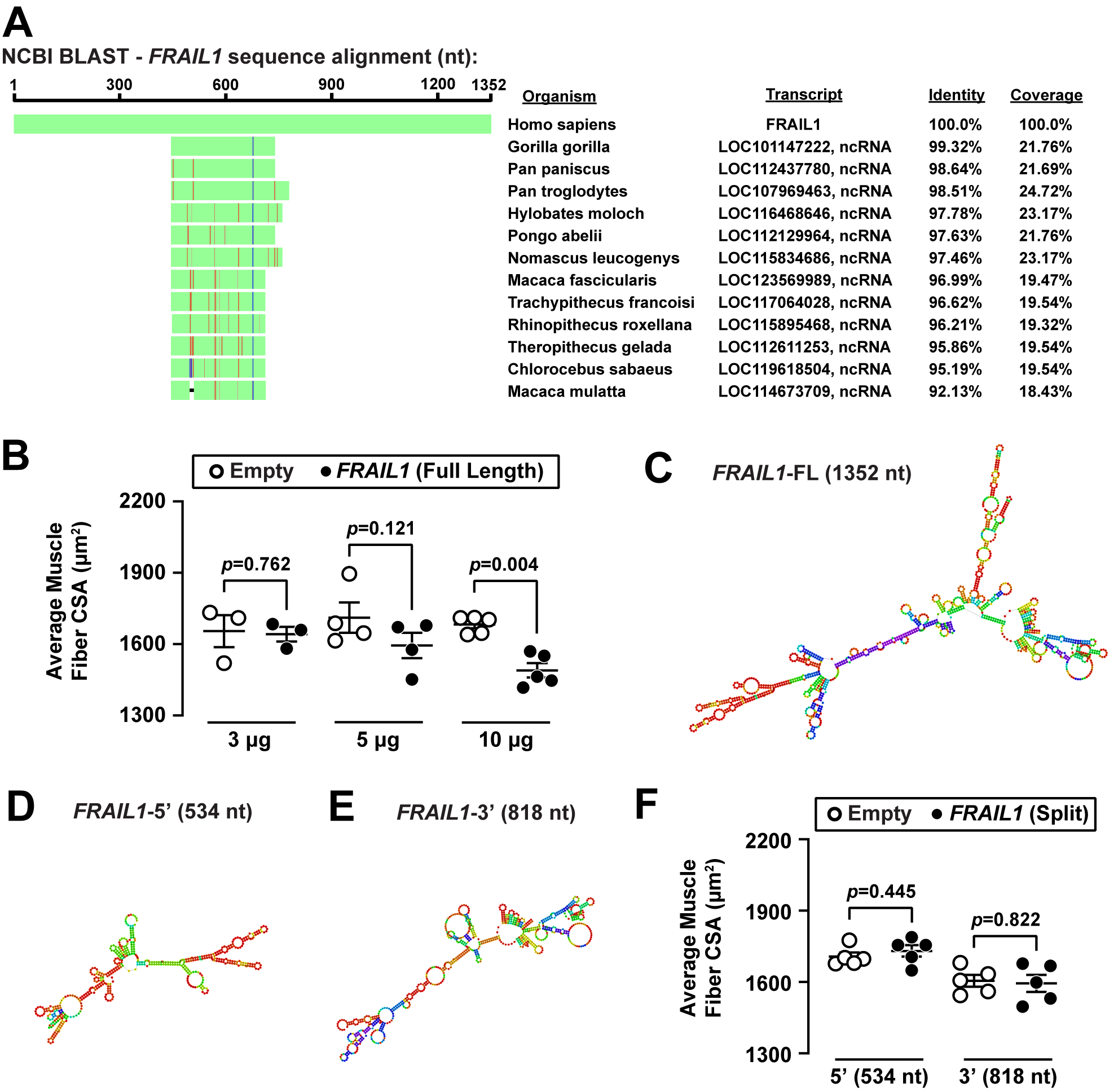

Supplement: Supplementary file 4 — Figure S4. [file ACEL-23-e14097-s002.tif]

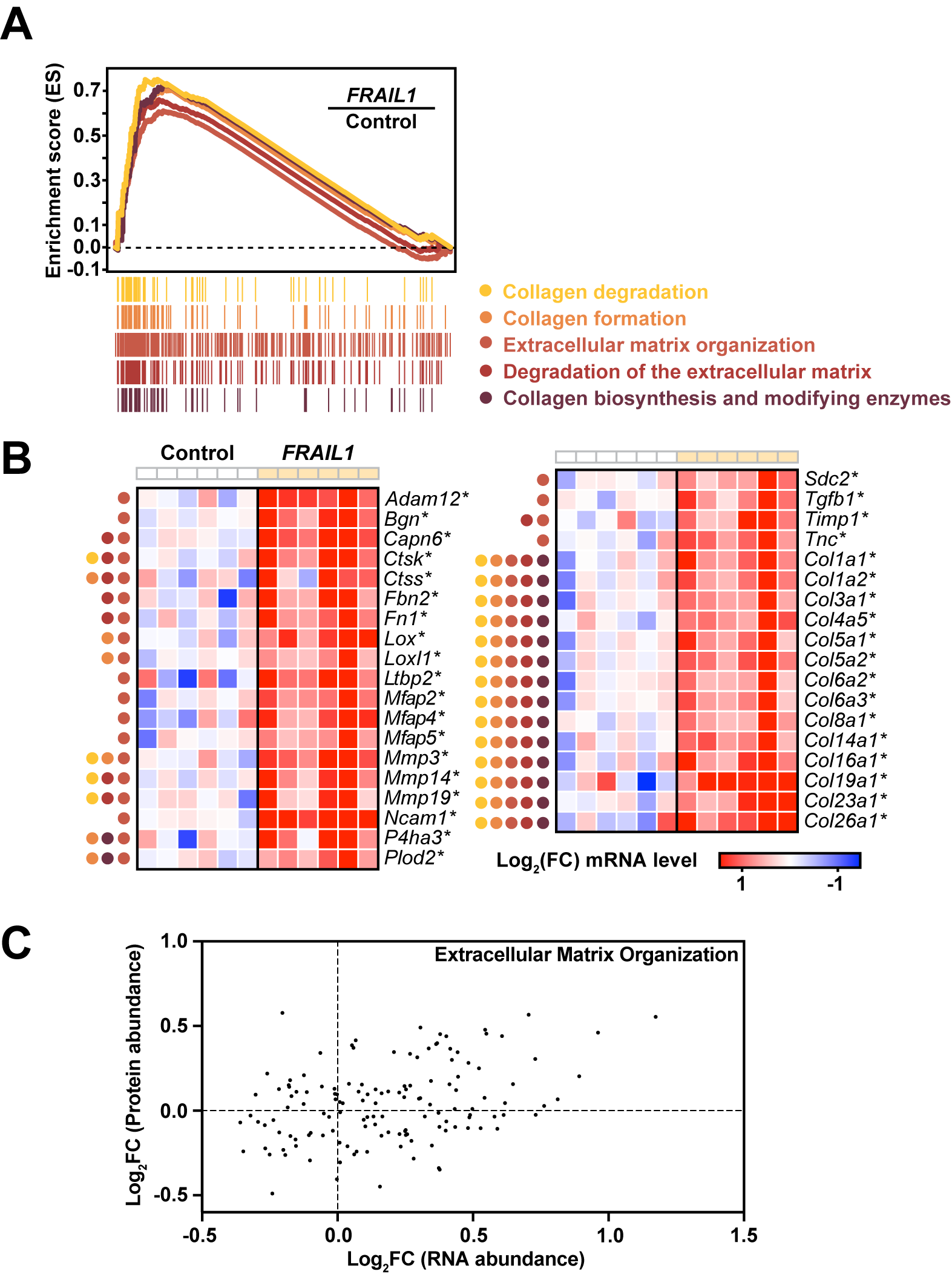

Supplement: Supplementary file 5 — Figure S5. [file ACEL-23-e14097-s004.tif]
